# Supplementary material for: Morphometric and Nanomechanical Features of Erythrocytes Characteristic of Early Pregnancy Loss
Source: Int J Mol Sci. 2022 Apr 19;23(9):4512. doi: 10.3390/ijms23094512 (PMC9103795; doi:10.3390/ijms23094512)
Supplement: Supplementary file 1 [file ijms-23-04512-s001.zip › ijms-1684698-supplementary.pdf]

Supplementary Material

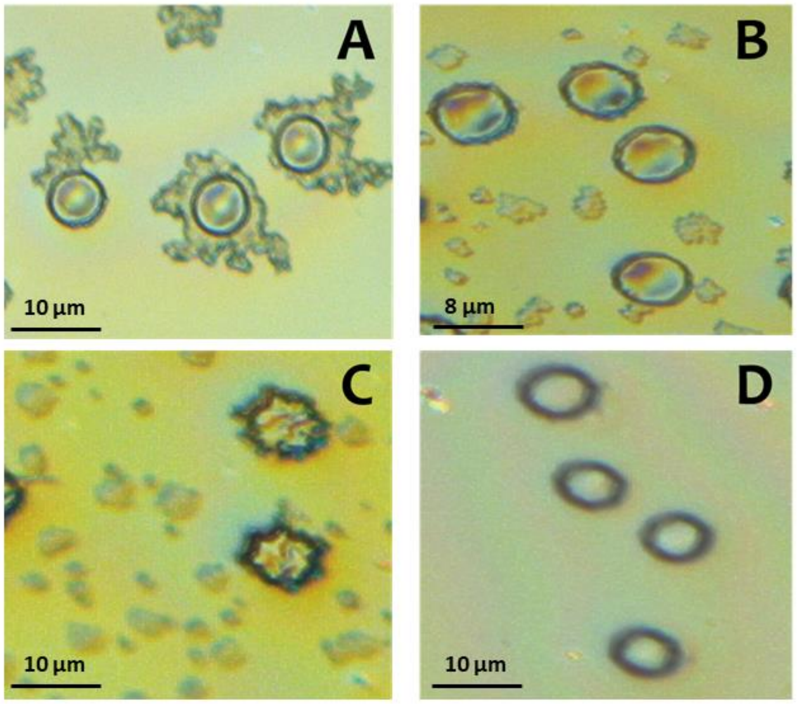

Figure S1. Representative optical microscopy images of four morphological types of erythrocytes observed in the process of their aging: (**A**) biconcave; (**B**) crenated; (**C**) spiculocytes and (**D**) spherocytes.

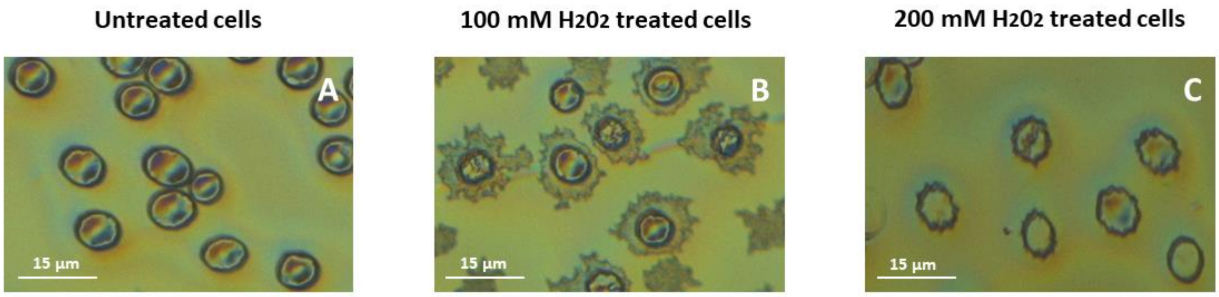

Figure S2. Representative images obtained by means of optical microscopy of untreated red blod cells (**A**) and cells treated with 100 mM (**B**) and 200 mM (**C**) H_2O_2.

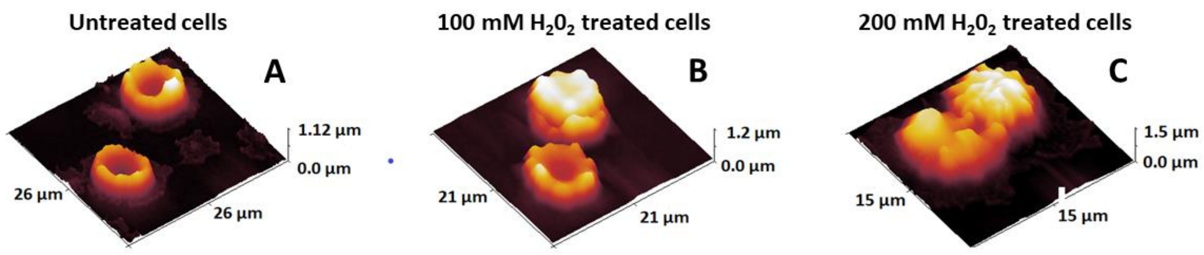

Figure S3. Representative 3D AFM images of untreated erythrocytes (**A**) and erythrocytes following exposure to H_2O_2 solution (**B**,**C**) .

Table S1. Pearson correlation coefficient, r, calculated for the pair of parameters Young's modulus and membrane roughness for RBCs of the studied groups of women.

Group	Young's Modulus, Ea	Membrane Roughness, R_{rms}
	Pearson's Correlation Coefficient, r	
NPC	−0.97	
PC	−0.98	
MS	−0.96	
